# Drone’s Angle-of-Arrival Estimation Using a Switched-Beam Antenna and Single-Channel Receiver †

**DOI:** 10.3390/s25082376

**Published:** 2025-04-09

**Authors:** Sumin Han, Byung-Jun Jang

**Affiliations:** Department of Electrical Engineering, Kookmin University, Seoul 02707, Republic of Korea; hsm2005x@kookmin.ac.kr

**Keywords:** unmanned aerial vehicles (UAVs), anti-drone sensors, angle-of-arrival (AoA), switched-beam antenna (SBA), single receiver, software-defined radio (SDR)

## Abstract

In this study, we propose a method to estimate the Angle-of-Arrival (AoA) of OFDM-based drone signals with wideband and burst characteristics using only a single-channel receiver and a switched-beam antenna. First, six circularly arranged directional antennas are time-division controlled using RF switches to measure the received power of each antenna. Next, the maximum beam pattern and the measured power of each antenna are synthesized in vector form, and the direction of the synthesized vector becomes the angle of arrival of the drone signal. To verify the proposed method, an experiment was conducted using the video signal of DJI Phantom 4 Pro with a bandwidth of 10 MHz. As a result, it was confirmed that stable angle-of-arrival estimation of drone video signals was possible with an average error of less than 5°. The proposed system has the advantage of being able to estimate the AoA of a drone with only a single receiver without the need for synchronization. Therefore, the proposed system is expected to be used as a low-cost, compact, and highly portable anti-drone system.

## 1. Introduction

The current applications of unmanned aerial vehicles (UAVs) and drones have diversified from aerial photography and smart agriculture to parcel delivery and infrastructure inspection. In addition, the military use of UAVs and drones has also increased, as observed in the Russian–Ukrainian war [1,2,3]. This trend has raised growing concerns about UAV and drone intrusions, leading to increased demand for reliable anti-drone systems [4]. Typically, anti-drone systems have been developed primarily as fixed installations to protect airports, military bases, and critical national infrastructure from unauthorized drones [5,6,7,8]. These systems are often high-performance devices capable of detecting multiple drones simultaneously, extracting unique features to identify the type and presence of each drone, and estimating their positions and flight trajectories for real-time tracking [9,10,11,12,13,14,15,16]. These systems are typically categorized into active and passive approaches. Active systems, such as radar-based methods [9,10,11,12], transmit probing signals and analyze their reflections, whereas passive systems, including RF-based, acoustic, and vision-based techniques, detect drones by receiving signals emitted by the drone [13,14,15,16,17].

As drones are increasingly used in various fields, the application areas of anti-drone systems have also expanded. In particular, there is a growing demand for compact and low-cost solutions that can be easily deployed in diverse environments. Among these, RF scanner-based architectures have emerged as a promising alternative to radar, offering lower complexity, reduced cost, and the ability to operate passively without emitting signals. In general, RF-based systems consist of two functional stages: detection, which identifies the presence and type of a drone, and localization, which estimates its position [4,15,16]. While considerable progress has been made in RF-based detection using AI-driven signal classification [18,19,20,21,22,23,24], accurate and real-time localization remains a key challenge, especially for lightweight systems. Among the various localization methods—such as time of arrival (TOA), time difference of arrival (TDOA), and received signal strength (RSS)—angle of arrival (AoA) has gained attention as a particularly promising solution [4,6,16]. Unlike TOA and TDOA, which require precise time synchronization, and RSS, which is highly sensitive to environmental factors, AoA estimation requires only an antenna array and does not depend on synchronized timing. This makes it suitable for real-time localization in complex environments such as urban areas.

AoA estimation determines the direction of incoming signals by analyzing the differences in phase or time of arrival across spatially separated antennas. Among various techniques, Phase Difference of Arrival (PDoA) is widely used, where the phase shift between signals received at different antennas is measured to infer the direction. This principle underlies many classical AoA methods such as MUSIC and ESPRIT, which assume either simultaneous sampling across multiple antennas or accurate knowledge of signal structure [25,26,27,28,29]. However, the way AoA estimation is implemented largely depends on the characteristics of the received signal. For example, in Bluetooth, each packet must use a continuous wave (CW) tone, which provides a stable reference for measuring the phase difference. To reduce hardware complexity, this allows AoA estimation using a single receiver by rapidly switching between antennas and applying PDoA across time-separated samples [25,26]. In contrast, Wi-Fi signals, which are typically based on OFDM, do not include an explicit CW tone like in Bluetooth. However, they embed known pilot symbols on specific subcarriers, which serve as reference points for channel and phase estimation. These pilots enable the extraction of Channel State Information (CSI), at each antenna, from which phase differences can be calculated. A summary of existing AoA estimation methods for anti-drone systems, including both phase- and power-based approaches, is presented in Table 1. As shown in Table 1, systems such as ArrayTrack [27] and SpotFi [28] have achieved good accuracy by exploiting the CSI reference signal of Wi-Fi and using multiple synchronized receivers, but at the expense of hardware complexity. For example, a six-antenna system would require six individual receivers, significantly increasing cost, complexity, power consumption, and system weight. Moreover, proper synchronization and calibration among these receivers is essential, further complicating system design. On the other hand, to address these issues, systems like Phaser [29] attempt to emulate phased arrays by rapidly switching among multiple antennas connected to a single receiver. This approach enables PDoA estimation without the need for multiple receivers. However, it suffers from phase drift due to the lack of simultaneous sampling, necessitating autocalibration. Even with compensation, Phaser [29] reported a mean angular error of approximately 20°, whereas systems with fully synchronized multi-receiver architectures achieved accuracy within 2°. Phaser [29] reported a mean angular error of approximately 20°, whereas systems with fully synchronized multi-receiver architectures achieved accuracy within 2°. Recently, two studies by Florio [30,31] have demonstrated that FPGA-based full-digital architectures can achieve fast and accurate AoA estimation. These systems implemented the entire AoA pipeline—including phase detection and angle computation—within reconfigurable hardware, enabling real-time processing in a memory-less configuration. Their results showed average errors of less than 2° and significantly reduced latency compared to software-based approaches such as MUSIC.

However, it is important to note that these methods were validated only on CW signals and not on unknown or burst-mode signals such as encrypted OFDM. Therefore, although these FPGA-based approaches are promising, applying them to signals with unstable or hidden phase references remains a challenge. This limitation becomes even more critical in the case of drone-generated OFDM signals. Drones commonly use OFDM schemes such as Wi-Fi to transmit video data [32,33]. These signals are typically wideband, transmitted in burst mode, and therefore discontinuous. Although OFDM includes subcarriers with CW-like pilots for synchronization, drone communication protocols are often proprietary or encrypted, making it difficult to identify or isolate a reliable phase reference. These characteristics pose significant challenges for applying traditional AoA techniques such as MUSIC and ESPRIT [27,28,29], which require stable reference signals for accurate phase-based estimation. In a related approach, ref. [34] attempted to estimate the AoA of drones by leveraging frequency-hopping spread spectrum (FHSS) signals. His method utilized phase-based algorithms to track the direction of arrival across rapidly changing carrier frequencies. However, FHSS signals—by nature—hop across wide frequency bands in short time intervals, and drone implementations often employ obfuscated hopping patterns and encryption. As a result, synchronizing phase measurements and tracking the frequency hops in real time remains technically challenging, limiting the applicability of the approach in practical drone detection scenarios. To address these limitations, several recent studies have bypassed phase-based estimation entirely and instead adopted received signal power as the key feature for AoA [35,36,37,38,39]. For instance, refs. [35,36] successfully estimated the AoA of Wi-Fi transmitters using a switched-beam antenna (SBA) by measuring the average signal power across beams. However, most of these systems do not operate in real time, as signal acquisition and processing are performed separately. In the context of drone signals, ref. [39] proposed a power-based AoA estimation system that rotates a directional antenna using a motorized platform. While this approach reduces the reliance on phase information and avoids the need for multiple receivers, it reported an average angular error of 12.2° and requires mechanical movement, which limits real-time performance and system durability.

**Table 1:** Summary of AoA estimation methods in prior work, including signal types, estimation techniques, and reported accuracy.

Reference	Signal Type	Estimation Method	Average Error (°)	Setup
ArrayTrack [27]	CSI-based	PDoA	7.4	Multi-receiver
SpotFi [28]	CSI-based	PDoA + MUSIC	<5	Multi-receiver
Phaser [29]	CSI-based	PDoA + Autocalibration	20/ 2	Single-receiver/ Multi-receiver
Batuhan Kaplan [34]	Drone’s FHSS	PDoA + MUSIC + Autocalibration	1.39	Multi-receiver
Phuc Nguyen [39]	Drone’s video	Power-based Rotating single antenna	12.2	Single-receiver
Antonello Florio [31]	CW	Phase interferometry (Full-digital)	0.34	Multi-receiver (4-ch FPGA)
This Work	Drones’ video	Power-based + Switched-beam Antenna (SBA)	<5	Single-receiver

In response to the aforementioned challenges, this study proposes a real-time anti-drone AoA estimation system using a single receiver and a switched-beam antenna with simple signal processing. Specifically, we use a simple RF switch to activate each antenna beam in a time-division manner, allowing the system to measure the received signal power sequentially and estimate the AoA of the drone. In this study, the system operates under a quasi-real-time constraint, computing a new AoA estimate every 1.2 ms based on the antenna switching cycle. While we did not formally evaluate strict real-time guarantees, the system demonstrates timely and continuous angle tracking. This interval was selected based on practical considerations related to signal acquisition and processing of the OFDM video signal. Our system can operate almost in near real time.

The core contribution of this study lies in the compact and cost-efficient architecture enabled by the use of a single-channel receiver. Unlike conventional AoA systems that require multiple synchronized receivers, complex calibration, and expensive RF front-ends [27,28], our design eliminates synchronization circuitry and reduces both hardware complexity and power consumption. Although the system is implemented using an SDR platform and GNURadio for flexibility in prototyping, the architecture itself is independent of these tools. It can be readily translated to lower-cost, embedded, or FPGA-based implementations for field deployment.

**Figure 1:**
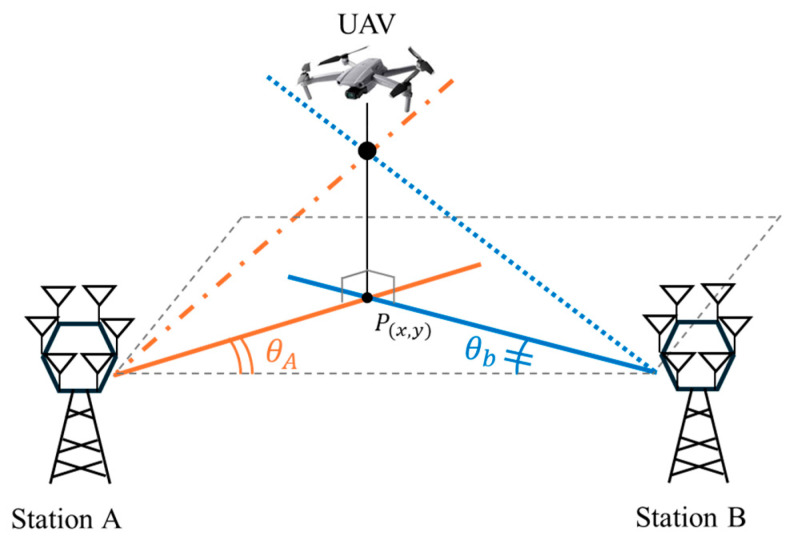
Estimation of the drone’s position using two RF scanners with angle-of-arrival capability.

In this study, we focus on developing and validating an efficient AoA estimation method, aiming to provide a practical foundation for RF scanner-based drone localization. Experimental results validate the effectiveness of the proposed method, demonstrating an average angular error of less than 5°, both for continuous wave (CW) signals and drone-generated OFDM video transmissions. This makes it a promising candidate for integration into low-cost, compact, and portable anti-drone systems. As shown in Figure 1, a typical AoA-based drone position estimation system involves two or more stationary devices that estimate the AoA of received signals. The suggested method can be used to infer the drone’s position by estimating the AoA of such a drone [8,9].

## 2. Proposed System Configuration and Operating Principle

**Figure 2:**
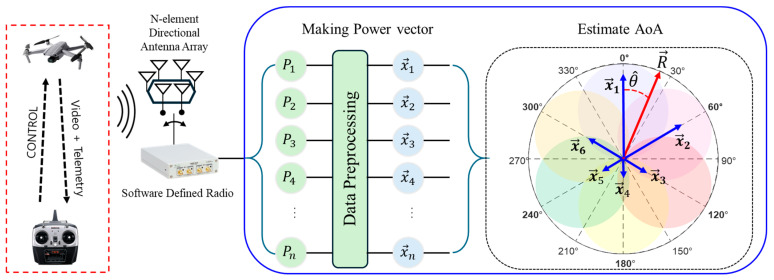
Proposed drone angle-of-arrival estimation system.

As shown in Figure 2, the proposed drone’s AoA estimation system consists of a single receiver implemented as a Software-defined Radio (SDR), a circular SBA consisting of six antennas, an RF switch with a fast-switching speed controlled by a microcontroller, and a signal processor performing AoA estimation.

First, the antenna receives an OFDM-based video signal emitted from a drone. In this process, the SP6T (single-pole 6-throw) switch operates with a switching cycle of 1.2 ms, so that the drone’s video signal is sequentially received by each antenna. Then, the signals received from the antennas are fed to the SDR, where frequency down-conversion is performed to generate in-phase (I) and quadrature-phase (Q) baseband signals for processing by the PC. During signal processing, the complex I/Q samples are separated according to the corresponding antenna. Next, the received power for each antenna is calculated to form a power vector. Then, this power vector is used to estimate the AoA of the drone. That is, multiple power measurements are performed within a single signal packet, and a power-based AoA estimation algorithm is applied to determine the direction in which the drone is approaching. Unlike conventional array-based approaches that rely on the phase difference in incoming signals across multiple antennas, this method uses only the measured power at each antenna. Therefore, as long as the power measurement is accurate, the signal’s modulation characteristics do not affect the AoA estimation.

Mathematically, the principle of operation can be described as follows. Six antennas are arranged in a circle, and the SP6T switch activates each antenna in turn at very short intervals of a few hundred microseconds. The SDR receives a continuous I/Q data stream but, with reference to the switching operation, these data are segmented and stored per antenna. Let $r_{n}\;\left(k\right)$ denote the signal received by the *n*-th antenna. Therefore, the signal received by each antenna can be expressed as

(1) $$r_{n}\left(k\right)=a_{n}\left(\theta \right)s\left(k\right)+w_{n}\left(k\right),n=1,\;2,\;\ldots ,\;6,$$

where k denotes the sample index, $a_{n}\left(\theta \right)$ is the beam-pattern gain (amplitude gain) of the n-th antenna in the θ direction, s(k) is the drone signal arriving at the antenna, and $w_{n}\left(k\right)$ is noise with a mean of zero and variance $\sigma^{2}$. If $M_{n}$ is the number of samples acquired during the active interval of the n-th antenna, the received power $\hat{P_{n}}$ at the n-th antenna can be computed in the time domain as

(2) $$\hat{P_{n}}=\frac{1}{M_{n}}\overset{M_{n}}{\underset{k=1}{\sum }}|r_{n}(k){|}^{2}$$

Now, if we can measure the power for each of the six antennas within a single continuous packet of the drone video signal, a power vector P is generated, which is expressed as

(3) $$\hat{P}={\left[\hat{P_{1}\;},\;\hat{P_{2}\;},\;\ldots ,\;\hat{P_{6}}\;\right]}^{T}$$

The main beam direction of each antenna $\phi_{n}$ (i.e., the angle corresponding to the maximum gain of the antenna) is determined in advance through measurement or simulation. Therefore, for the six antennas, the directions {$\phi_{1},\;\phi_{2},\;\ldots ,\;\phi_{6}\}$ and the corresponding power measurements $\left\{P_{1},\;P_{2},\;\ldots ,\;P_{6}\right\}$ can be expressed as ($\phi_{n},\;\hat{P_{n}})$. Thus,

(4) $$u\left(\phi_{n}\right)={\left[\cos\left(\phi_{n}\right),\sin\left(\phi_{n}\right)\right]}^{T}$$

(5) $${\vec{x}}_{n}=\hat{P_{n}}\;\cdot \;u\left(\phi_{n}\right)$$

where $u\left(\phi_{n}\right)$ denotes a unit vector in the 2-dimensional plane at angle $\phi_{n}$. Now, multiplying $u\left(\phi_{n}\right)$ by a power P define a vector $\vec{x_{n}}$. As shown in Figure 1, the vector sum of these antenna power vectors yields the composite vector R as

(6) $$R=\overset{6}{\underset{n=1}{\sum }}{\vec{x}}_{n}=\overset{6}{\underset{n=1}{\sum }}\left(\hat{P_{n}}\;\cdot \;u\left(\phi_{n}\right)\right)$$

The composite vector R can be represented by its magnitude |R| and direction ∠R. Because the vector-sum operation mainly reflects the direction of the antenna with relatively high received power (i.e., the antenna that strongly detects the actual drone signal), the direction of R tends to form near the true angle of arrival $\theta_{ture}$. As a result, the drone’s angle of arrival $\hat{\theta }$ can be found as

(7) $$\hat{\theta }=∠\left(\max\left(R\right)\right)$$

This approach has the advantage of estimating the AoA using only the power measurements from antennas with different beam patterns, so there is no need for multi-channel synchronization or phase estimation. However, if the beamwidths are too wide or multipath occurs, the directionality of R may be weakened, which may reduce the AoA estimation accuracy. However, since drones operate in the sky and usually maintain a clear line of sight, this problem is unlikely to occur.

## 3. System Implementation and Signal Processing

### 3.1. Hardware Configuration

**Figure 3:**
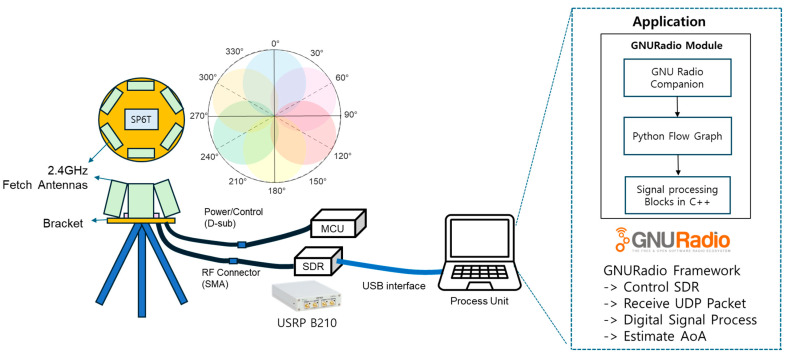
Hardware and signal processing framework of the proposed switched-beam antenna system: SDR control, packet reception, and AoA estimation implemented via GNU Radio over USB interface.

The hardware setup used in this study is shown in Figure 3. The circular array antenna is designed to be fixed using a tripod. The USRP B210 SDR is adopted to receive signals in the 2.4 GHz frequency band, which is commonly used for video transmission from drones [40]. The six directional antennas are arranged in a circle so that their maximum radiation directions are 60° apart. The elevation angle can also be mechanically adjusted to detect drones approaching from above.

A key design principle of our system is the use of a single RF receiver, which minimizes hardware complexity compared to conventional AoA systems that rely on multiple synchronized receivers. This design is made possible by incorporating a switching mechanism that sequentially activates each antenna element, allowing directional signal power measurements to be collected over time through a single receiver.

For prototyping and experimental validation, we adopted a Software-defined Radio (SDR) receiver due to its high flexibility, seamless integration with host computers, and compatibility with artificial intelligence (AI)-based signal processing tools. Although SDRs are sometimes considered more expensive or complex than FPGA-based systems, they are widely adopted in research environments due to their rapid development capabilities. In this study, the SDR enabled us to efficiently implement and test the proposed signal acquisition and beam-switching mechanisms. It is worth noting that the proposed system architecture is not limited to SDR-based implementations. Once the overall design is validated, the same structure can be readily translated to cost-effective FPGA or embedded systems, which are more suitable for real-time field-deployable anti-drone applications. This flexibility makes our approach scalable and adaptable to a wide range of operational environments. To process the received signals and estimate the AoA in real time, the entire signal acquisition and signal processing pipeline was implemented using GNURadio on a standard laptop. As shown in Figure 3, GNU Radio controls the SDR via USB and handles both packet reception and real-time processing. The AoA computation module was developed in Python (version 3.7 or higher) with C++ bindings. This approach allows direction estimates to be computed every 1.2 ms. This demonstrates real-time capability without requiring any custom HDL or dedicated hardware.

**Figure 4:**
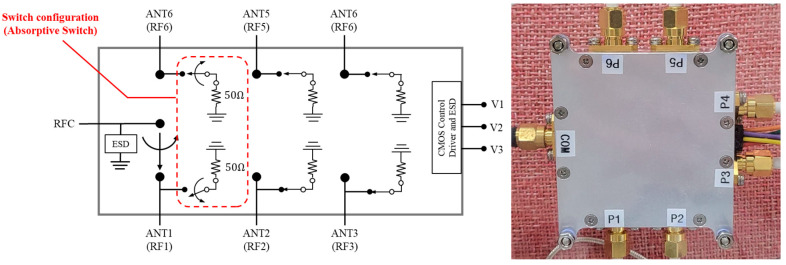
Designed SP6T antenna switch block diagram and photograph.

**Figure 5:**
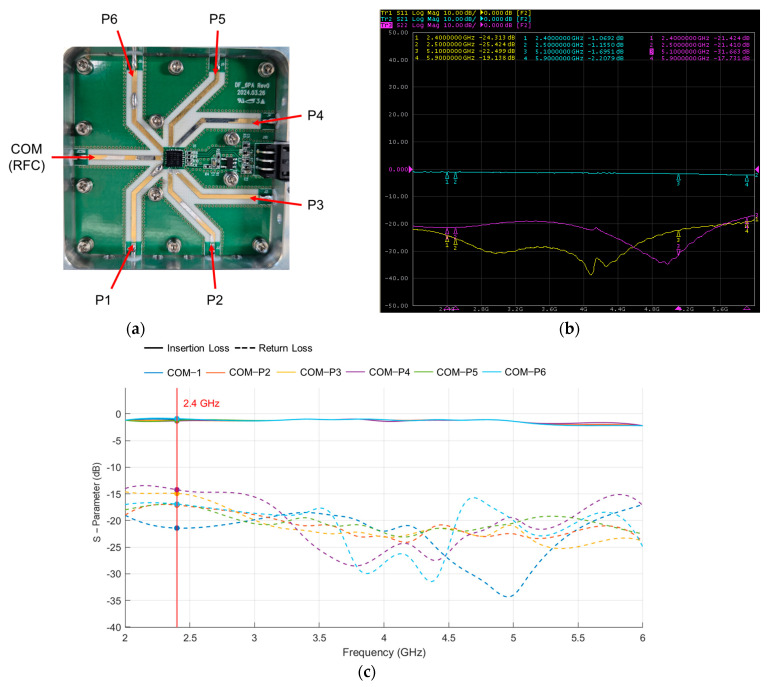
Measured S-parameters of the SP6T switch using a Vector Network Analyzer (VNA): (**a**) photograph of the internal SP6T switch hardware; (**b**) measured S-parameters for COM–P1 using VNA; (**c**) detailed plot showing insertion loss and return loss characteristics for all six antenna ports across the 2–6 GHz frequency band.

The SP6T switch, implemented using Peregrine Semiconductor’s UltraCMOS PE426462 RF switch IC [41], is placed in the center of the antenna array to sequentially activate one antenna at a time. The switch features high isolation (40 dB at 2 GHz) and low insertion loss (0.9 dB at 2 GHz), making it well suited for multi-antenna configurations of our system. The switch operates in an absorptive switch configuration. The selected port passes the RF signal, while all unselected ports are terminated with a 50 Ω termination load. This termination effectively absorbs residual signals and prevents them from being reflected back toward the antennas, thereby minimizing undesired coupling or interference that could degrade the accuracy of AoA estimation. The configuration and internal structure of the SP6T RF switch are illustrated in Figure 4. In addition, Figure 5 presents the internal PCB layout of the SP6T switch along with measured S-parameters obtained using a Vector Network Analyzer (VNA) to evaluate the switch’s performance in terms of insertion loss and return loss across the 2–6 GHz band. The measurement includes the insertion loss at each of the six antenna ports (COM–P1 to COM–P6) as well as the return loss characteristics of all six ports. These results provide quantitative evidence of low-loss signal transmission and good impedance matching around the 2.4 GHz operating band.

The SP6T RF switch consists of a power/control connector (D-Sub) and an SMA RF connector. The power/control connector is connected to a microcontroller unit (MCU) that controls the switch, and the RF connector is connected to the USRP B210 SDR that down-converts the received 2.4 GHz radio signals. The down-converted in-phase (I) and quadrature-phase (Q) data are then captured on the PC, where the signal processing unit performs AoA estimation.

**Figure 6:**
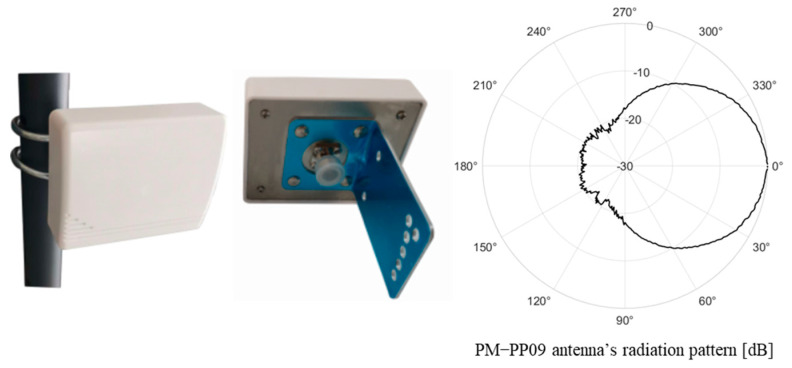
PM-PP09 antenna and its radiation pattern.

In this study, we used commercially available PM-PP09 microstrip array antennas. This antenna operates in the 2.400–2.483 GHz and provides a directional gain of about 9 dBi. Figure 6 shows the horizontal gain pattern of the PM-PP09 antenna. As shown in Figure 6, the 3 dB beamwidth is about 60°, and the gain decreases at angles outside this beamwidth.

In conventional phase-based AoA estimation systems, mutual coupling and hardware-induced artifacts often introduce significant angle estimation errors, especially when multiple receivers are used simultaneously. However, the system proposed in this study avoids these issues in two ways. First, the AoA estimation method employed here is based on received signal power rather than phase difference, thereby eliminating the sensitivity to phase distortions commonly introduced by mutual coupling or circuit mismatches. Second, the six antennas are arranged symmetrically in a circular formation, and only one antenna is active at a time through the SP6T RF switch. From the perspective of each receiving instance, all inactive antennas form a passive and symmetrically placed environment around the active one. This symmetry ensures that any mutual coupling effects are spatially uniform and cancel out across antenna switches, effectively making their influence constant and negligible for power-based estimation.

### 3.2. Singal Processing Unit Implementation

**Figure 7:**
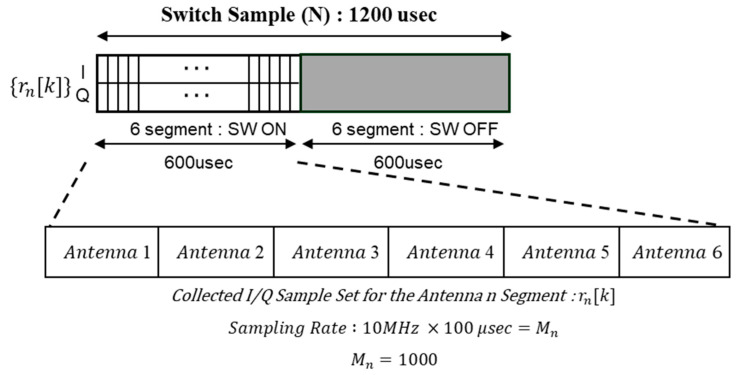
Antenna switching procedure and signal processing of angle-of-arrival estimation.

An important consideration in implementing the signal processing unit is the synchronization between antenna switching and data acquisition. In a typical general-purpose PC environment without a real-time operating system, it is very difficult to achieve microsecond-level switching and scheduling. Because the SDR device transmits high-bandwidth I/Q data over UDP, the data arrival time and order can be irregular, and there is an inherent delay between the internal transmission buffer of the SDR and the network buffer of the PC. As a result, it becomes difficult to maintain perfect synchronization between the RF switch operation and I/Q data indexing. When the video signal packet of the drone is very short (on the order of a few hundred microseconds), the timing constraints become even tighter because all antennas must be activated sequentially within that limited time. The existing solution is to use a multi-channel receiver to collect signals from all antennas simultaneously. However, this approach is not compatible with the goals of being low cost and lightweight. Therefore, in this study, we utilize a low-cost Arduino microcontroller to periodically operate the SP6T switch at microsecond intervals, and the PC asynchronously collects I/Q data from the SDR. In asynchronous acquisition, the order of antenna activation is not immediately clear, but by inserting short idle periods between the switch on/off intervals, the active segments of each antenna can be identified. This process is shown in Figure 7. As shown in Figure 7, if the packet length of the drone video signal is greater than 1.2 ms, the power value in each segment will be the power received by each antenna.

The switching period is determined by considering the drone’s video signal packet duration, bandwidth, number of antennas, and the desired AoA estimation update rate. In this study, six antennas are sequentially activated for 100 μs each, making the total sampling time 600 μs. Then, an idle interval of 600 μs is added to distinguish the antennas, making the total switching period 1.2 ms, as shown in Figure 7. At a sampling rate of 10 MHz, each 100 μs active interval of each antenna corresponds to 1000 I/Q samples (10 MHz × 100 μs = 1000 samples). Therefore, approximately 1000 complex samples are acquired during the active window of each antenna. From these samples, the average power of the nth antenna ($\hat{P_{n}}$) is calculated. Aggregating these power measurements into a power vector allows us to estimate the AoA of the drone. By repeating this process every 1.2 ms, we can track the moving drone in quasi real time.

The switching period is determined by considering the drone’s video signal packet duration, bandwidth, number of antennas, and the desired AoA estimation update rate. In this study, six antennas are sequentially activated for 100 μs each, making the total sampling time 600 μs. Then, an idle interval of 600 μs is added to distinguish the antennas, making the total switching period 1.2 ms, as shown in Figure 5. At a sampling rate of 10 MHz, each 100 μs active interval of each antenna corresponds to 1000 I/Q samples (10 MHz × 100 μs = 1000 samples). Therefore, approximately 1000 complex samples are acquired during the active window of each antenna. From these samples, the average power of the nth antenna ($\hat{P_{n}}$) is calculated. Aggregating these power measurements into a power vector allows us to estimate the AoA of the drone. By repeating this process every 1.2 ms, we can track the moving drone in real time.

## 4. Experimental Results and Discussion

**Figure 8:**
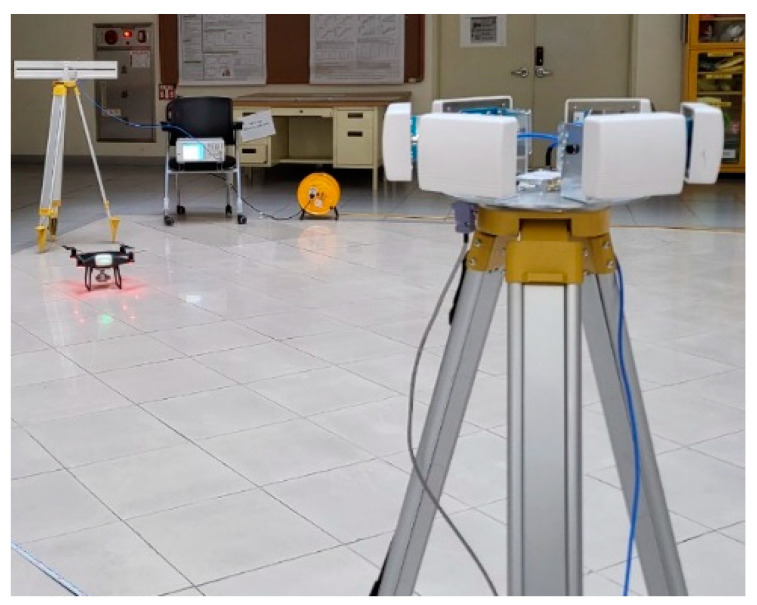
Experiment setup using switched-beam antenna and DJI Phantom 4.

**Figure 9:**
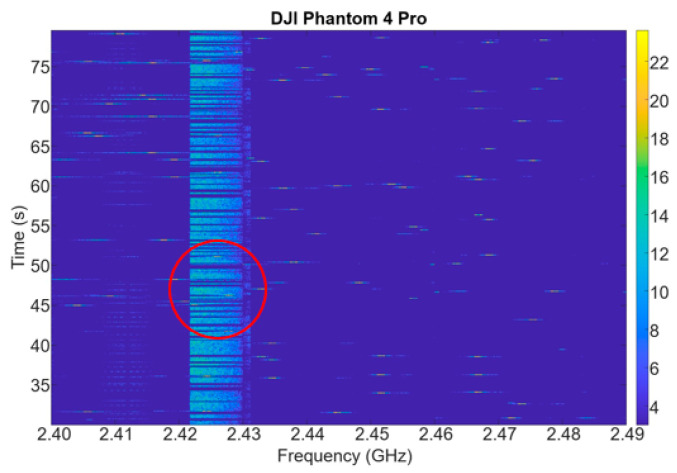
Experiment results showing the spectrogram monitored via the USRP B210 SDR.

To verify the effectiveness of the proposed drone’s AoA estimation method using a switched-beam antenna and single-channel SDR, an experiment was conducted as shown in Figure 8. The experiment was conducted in the hall of Kookmin University, Seoul, the Republic of Korea, and because the hall is very large (12 m × 20 m × 30 m), we confirmed that the results were almost the same as outdoors, even though it was indoors. The video transmission signal of the DJI Phantom 4 Pro drone (DJI, Shenzhen, China) was used. In order to evaluate the proposed power-based AoA system, the sampling rate and antenna switching period were configured prior to measurement. The center frequency and sampling rate of the SDR were determined as follows. First, in order to identify the exact center frequency of the video signal of the drone, an omni-directional antenna was connected to the SDR, and the entire 2.4 GHz ISM band was scanned. As a result of the experiment, it was confirmed that the DJI Phantom 4 Pro used in the experiment transmitted a video signal with a bandwidth of approximately 10 MHz and the frequency did not change, as shown in Figure 9. Next, the sampling rate of the SDR was determined based on the bandwidth of the video signal. Since the proposed power-based AoA estimation is more important to measure the power between antennas and know the difference than to perform detailed signal demodulation, oversampling is not necessary [36]. In other words, the sampling rate at the level of the signal bandwidth (~10 MHz) is sufficient. Based on this observation, each of the six antennas was activated for 100 µs (600 µs in total), followed by a 600 µs idle interval, resulting in a total switching cycle of 1.2 ms. The AoA was estimated once per cycle.

With this configuration, a baseline experiment was first conducted using a CW signal at the same center frequency (2.44 GHz) prior to testing with drone-emitted OFDM video signals. This initial test was designed to evaluate the system’s fundamental direction-finding accuracy under ideal conditions. The CW signal was generated using a signal generator and transmitted toward the antenna array. The proposed power-based AoA estimation algorithm was applied, and the resulting angular error remained within 5°, confirming the system’s baseline performance. Next, using the same configuration, we applied the method to the video transmission signal of the DJI Phantom 4 Pro drone.

**Figure 10:**
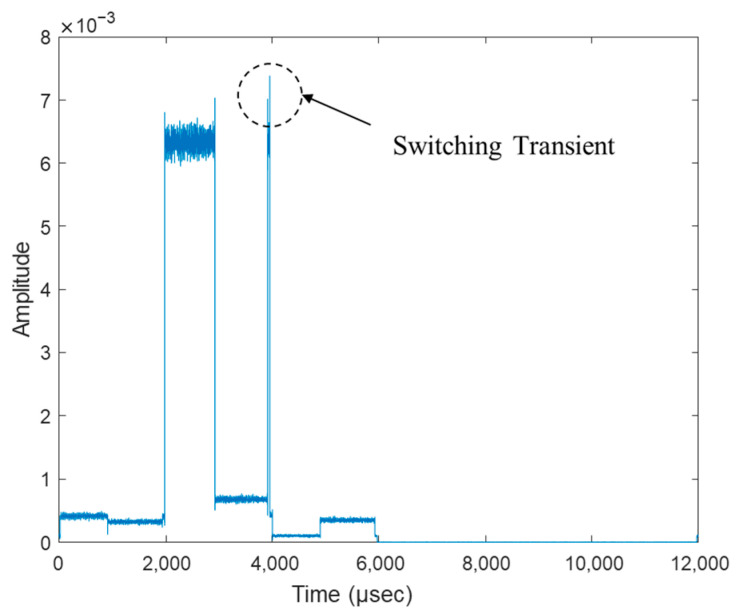
Experiment results: OFDM signal received from the drone using SBA.

Figure 10 shows an example of the collected I/Q data when the drone was positioned in front of antenna 3. Each 100 μs segment contained approximately 1000 samples, with signal magnitudes varying across antenna beams due to directional differences—most notably, antenna 3 exhibited the highest amplitude. During both CW and OFDM experiments, brief signal spikes known as switching transients were observed during antenna switching events. These occurred due to hardware switching delays and UDP-based transmission jitter. To mitigate their effects, a filtering technique was applied that calculates the median of the top 80% of samples within each active segment. This approach produced more stable power measurements and improved AoA estimation accuracy. Overall, this process stabilizes the power calculation per antenna, improving the reliability of AoA estimation for drone signals.

**Figure 11:**
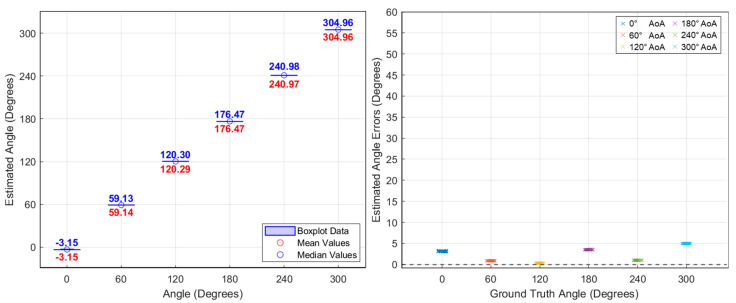
Angle-of-arrival estimation experiment results for CW signals using a signal generator.

**Figure 12:**
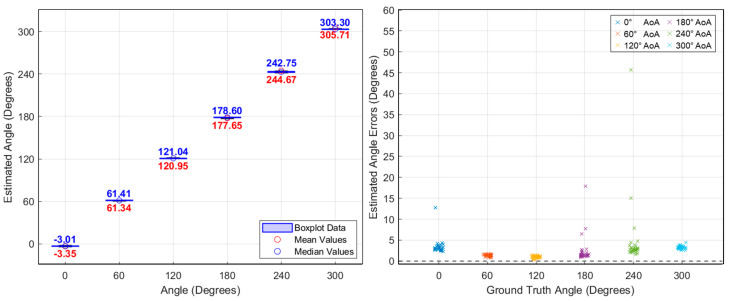
Angle-of-arrival estimation experiment results for DJI Phantom 4 Pro OFDM video signals.

**Figure 13:**
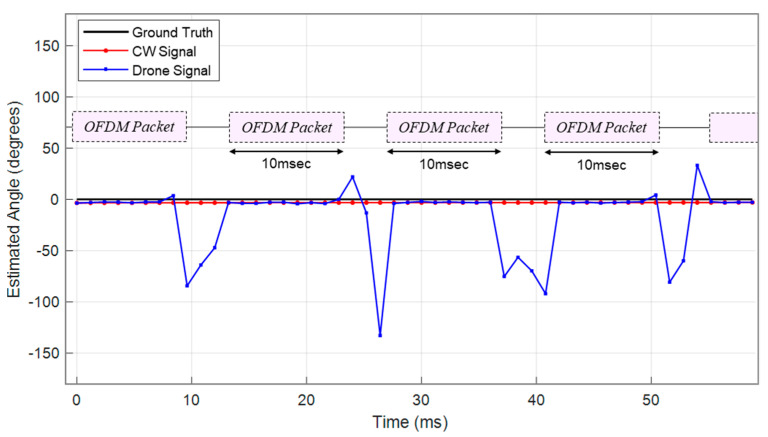
Real-time AoA estimation performance under CW and drone signal conditions. The estimations are referenced to 0° and computed every 1.2 ms based on the switching cycle.

As previously mentioned, angle estimates were computed every 1.2 ms, and additional tests were performed by rotating the signal source by 60°. As shown in Figure 11, the average AoA estimation error for the CW signal in the baseline experiment was within approximately 5°. For comparison, the results for the drone’s video signal are presented in Figure 12, where the average AoA estimation error also remained less than 5°. Figure 13 shows the AoA estimation results over time. While CW signals show consistently stable estimates, the drone’s OFDM-based video signals occasionally produce estimation spikes due to packet loss. As shown, AoA estimation remains stable during the 10 ms duration of each OFDM packet but fails when the signal disappears. Nevertheless, accurate direction finding was consistently demonstrated whenever the OFDM signal was present.

Although our experiment was conducted on the ground indoors, actual anti-drone systems are typically deployed on elevated structures such as rooftops or towers, where the impact of ground-reflected multipath signals is generally reduced. While we acknowledge that multipath effects can influence power-based AoA estimation, they were not the primary focus of this study. Instead, we aimed to evaluate the feasibility of artifact-agnostic estimation using a simple switched-beam approach. Related studies, such as Maddio et al. [38], proposed hardware-level mitigation using circularly polarized antennas and gain calibration matrices. Similarly, Florio et al. [42] introduced α-matrix–based linear correction techniques to enhance robustness in lightweight AoA systems. Adapting such techniques to power-based architectures remains an area for future exploration.

## 5. Conclusions

This study proposes and implements a system that can estimate the AoA of a drone using a single-channel receiver and a switched-beam antenna, eliminating the need for a multi-channel receiver. By adopting a power-based processing approach, the system demonstrates stable AoA estimation for wideband, discontinuous, and burst-mode OFDM signals. The experimental results show an average accuracy within 5° for both CW signals and video transmission of DJI Phantom 4 Pro. These results verify that power-based drone direction finding can be realized with a single receiver, paving the way for easy integration into existing drone monitoring and identification systems. A comparison with existing AoA estimation systems is summarized in Table 1, which highlights the trade-offs in accuracy, complexity, and hardware configuration across various approaches. Notably, while systems such as SpotFi and ArrayTrack achieve similar or slightly higher accuracy, they rely on multiple synchronized receivers and CSI processing. The Phaser system, though single-receiver based, suffers from phase drift, requiring autocalibration and yielding an error up to 20°. Our approach, by contrast, achieves competitive accuracy without the need for synchronization, CSI, or mechanical movement, using only a single receiver and simple switched-beam control. These results confirm that power-based AoA estimation is a viable approach for drone direction finding with minimal hardware complexity. The proposed method offers a practical foundation for low-cost, compact, and portable anti-drone systems.

## Data Availability

The data presented in this study are available at [https://github.com/hsm2012x/Sensors_DroneDF/tree/main].

## Abbreviations

The following abbreviations are used in this manuscript:

AOA	Angle of Arrival
UAV	Unmanned Aerial Vehicle
ToA	Time of Arrival
TDoA	Time Difference of Arrival
RSS	Received Signal Strength
PDoA	Phase Difference of Arrival
MUSIC	Multiple Signal Classification
ESPRIT	Estimation of Signal Parameters via Rotational Invariance Technique
CSI	Channel State Information
SDR	Software-defined Radio
FPGA	Field-programmable Gate Array
OFDM	Orthogonal Frequency Division Multiplexing
FHSS	Frequency Hopping Spread Spectrum
SBA	Switched-beam Antenna
RF	Radio Frequency
CW	Continuous Wave
SP6T	Single-pole 6-throw
